# From ashes to evidence: A study on the alterations in bloodstain patterns in high heat environments and post‐fire scenes

**DOI:** 10.1111/1556-4029.15689

**Published:** 2024-12-19

**Authors:** Zack Kowalske, Abdulrahman Oleiwi, Graham Williams

**Affiliations:** ^1^ School of Health, Education, Policing and Sciences University of Staffordshire Stoke‐on‐Trent UK; ^2^ Roswell Police Department Crime Scene Investigations Unit Roswell Georgia USA; ^3^ Centre for Biomedicine, Hull York Medical School University of Hull Hull UK

**Keywords:** angle of impact, arson investigations, bloodstain pattern analysis, drip stain, fire

## Abstract

Fire is often used to conceal or destroy evidence of violent crimes, making it essential to understand how fire environments affect forensic evidence, particularly bloodstain patterns. This study investigates the impact of high heat environments and fire on the morphology and analysis of bloodstain patterns. Using controlled fire exposure, bloodstains were analyzed pre‐ and post‐fire exposure on various substrates, including glass, painted drywall, and painted plywood. Measurements of angle of impact (AOI) and area of origin (AOO) were conducted using Faro Zone 3D Expert software. Despite physical alterations due to extreme temperature exposure, certain characteristics of the original bloodstains persisted. AOI calculations showed minimal deviation between pre‐ and post‐fire measurements, with standard deviations generally under two degrees. AOO estimations also demonstrated no substantial statistical differences between pre‐ and post‐fire data. The study confirms that bloodstain patterns retain observable traits despite exposure to high heat conditions, supporting the reliability of BPA in fire‐affected scenarios. These findings enhance the understanding of bloodstain behavior in fire environments, aiding forensic investigations in accurately analyzing bloodstain patterns in cases involving fire or high‐temperature conditions.

**Highlights**

- Bloodstains can remain resistant to exposure to high temperatures and fire exposure.
- Edge characteristics and original stain parameters can still be measured after fire exposure.
- Morphological characteristics of blood exposed to high temperature can aid in estimating heat source direction.
- Traditional reconstructive formulas in BPA demonstrate reliability in fire‐exposed environments.

## 1 INTRODUCTION

Fire has long been used by criminals as a tool to conceal or destroy acts of violence [1]. Within recent history, examples include a 2017 Arkansas man who used a home's natural gas to set fire to the residence in an attempt to conceal the death of a two‐year‐old infant [2] or a California man who in 2014 murdered his wife only to set fire to the residence in an attempt to destroy the crime scene [3]. Many more examples can be cited just by simply searching news records. With the use of fire as a tool to destroy evidence of violence, it is crucial to understand the influence of fire environments on potential evidence. Bloodstain pattern analysis (BPA) can aid in understanding aspects of incident scenes that exhibit bloodstains, including those affected by environmental variables and conditions. Key components in BPA are the geometric analysis of bloodstains to understand the flight and origin of the liquid blood that created the stain patterns. Bloodstains can undergo alterations due to environmental, atmospheric, or human and animal interference factors, leading to potential discrepancies in analysis [4, 5]. Reliable estimations require accurate measurements of stain dimensions, contingent on the blood's interaction with the environment during and after deposition.

The destructive properties of intentional fire setting can lead to the loss or alteration of pattern evidence by substrate consumption or disfigurement, or the concealment of bloodstain patterns by soot and debris. These effects are just the ones attributed to the fire event itself; fire suppression efforts add to the alterations, for example, dilution by water or foam and the dismantling of surfaces during overhaul efforts. In a study examining arson‐related homicides in the United States between 1985 and 1994, a significant proportion (46.6%) of cases in which the victim exhibited a cause of death which would have yielded bloodstain evidence; gunshot (20.3%), blunt force (14.1%), stabbing (20.3%), and cutting (1.7%) [6]. Further, as it relates to using fire to conceal the crime, it was found that 66% of the victims received burn injuries post‐mortem [6].

Directly related to the examination of bloodstains in high heat environments or environments that pose abnormally high temperatures, Brady and Tigmo (2002) found that elevated ambient temperature alone had little impact on stain sizes [7]. Larkin and Banks (2013) observed that liquid blood when subjected to heated surface temperatures (40°–251°C) demonstrated steady alterations to stain diameters as boiling points were reached with stable evaporation taking place [8]. Understanding the physical appearance and distribution effects that heated surfaces have on bloodstain patterns is critical to the correct analysis of those patterns. However, this is only of aid when the bloodstain pattern is visible to the examiner. Vineyard et al., (2019) studied the reliability of blood detection chemicals on charred wood with bloodstain patterns, finding that of the 96 samples only 4% provided a positive reaction for the presence of blood [9]. Further work by ATF's Fire Research Laboratory (2009) concluded that blood detection chemicals yielded no results in areas of heavy fire damage but provided positive results in areas less burned [10]. Where previous research has examined the detection of post‐fire bloodstains or the alterations due to heated surfaces, this study explores the uncertainties associated with the estimation of the angle of impact (AOI) and area of origin (AOO) of bloodstain patterns in high heat and fire environments [11]. For the purpose of this study high heat refers to the studied temperature range between 60°–398°C.

## 2 MATERIALS AND METHODS

### 2.1 Blood source

Widespread research in BPA has laid the foundation for animal blood to be acknowledged as an adequate substitute for human blood in research and education [12, 13, 14, 15]. In this study, equine blood, preserved with citrate, was acquired from HemoStat Laboratories (Dixon, CA). Equine blood was selected over human blood due to its ease of commercial procurement, reduced risk of human bloodborne pathogen exposure, and the avoidance of ethical concerns related to the utilization of human blood. Citrate was incorporated as an anticoagulant preservative to maintain the blood's longevity and consistency for prolonged research. Previous research validates that the use of anticoagulants exerts minimal influence on the characteristics of the resultant bloodstains and patterns, ensuring that the preserved blood remains usable for a period of up to 14 days [14, 15, 16]. The authors recognize that a limitation of this study is the lack of existing research literature on the accuracy of using equine blood when exposed to high temperatures. The blood samples were stored in refrigeration and were used within 14 days after the initial blood draw collection. Upon receiving the bloodstock package, the stock was agitated to ensure homogeneity, and then the volume was divided into 5 mL samples in plastic tubes. These samples were then refrigerated for storage. Prior to conducting experiments, the necessary volume of blood was removed and subjected to manual agitation to ensure homogeneity. Afterward, the samples were heated to 37°C using a water bath.

### 2.2 Single droplet generation

Experimentation was conducted in the climate‐controlled setting of the Roswell Police Department's Forensic Science Laboratory. Three common household construction materials were used as target surfaces: glass, painted drywall, and painted plywood. The target samples consisted of 60cmx60cm panels of painted plywood and drywall, as well as pyrex high temperature glass sheets. The wood and drywall panels were painted using a single coat of Kilz All Purpose Interior and Exterior Primer in white, followed by two coats of Behr Premium Plus Ultra White Semi‐Gloss Enamel paint. After allowing the painted surfaces to cure for at least 5 days, the target sheets were positioned to the selected vertical angle and kept in position with rubber chocks, with the angles verified using a Leica Disto inclinometer (Leica Geosystems AG, Germany). The prepared blood was drawn into a BrandZig 3 mL syringe with a 1″ 23 gauge needle and positioned 1 m above the target in a laboratory bench clamp. Sets of 10 drops at known angles of impact of 90°, 60°, 45°, and 30° were deposited onto the target. The plunger was slowly depressed at an even rate until the mass of the blood drop detached from the syringe. This allowed for consistency in mass size. The syringe was replaced between angle sets to diminish the buildup of blood material that would alter the initial drop size. After deposition onto the target, the resulting stains were then photographed with scales.

Once the pre‐fire documentation was completed, all stains were then subjected to direct fire exposure. This was accomplished by placing the target samples onto an exterior concrete platform to mitigate fire hazards caused by combustion. In addition to the burn environment, a fire extinguisher operated by a trained public safety officer was on standby with the research materials. A 16 oz. Bernzomatic liquid propane torch was ignited and used to apply direct fire application to the stains. Direct fire exposure was applied for approximately 30 s or until the combustion of the target material began. During this application, a Ryobi Infrared Thermometer was used to measure the surface temperatures of the target samples with a recorded range between 315 and 398°C. The fire was then extinguished and the stains photographed again with metric scales.

### 2.3 Impact pattern generation

The impact patterns generated were conducted in the same laboratory environment. The wood and drywall target sheets were mounted onto a wall within an area designated for stain pattern generation. The stain patterns were created utilizing a manual striking method, in which a blood‐soaked sponge, resting on an elevated ramp at a 45° angle, 1 m in height, and one‐meter distance from the center of the target area was manually struck with a 2.7 kg masonry brick (measuring 10 cm × 19.5 cm × 4 cm). This action created the impact stain patterns on the target surface. After the pattern was generated on the surface, the patterns were digitally photographed with scales. This process was repeated to obtain a sample size of 10 impact patterns.

**FIGURE 1:**
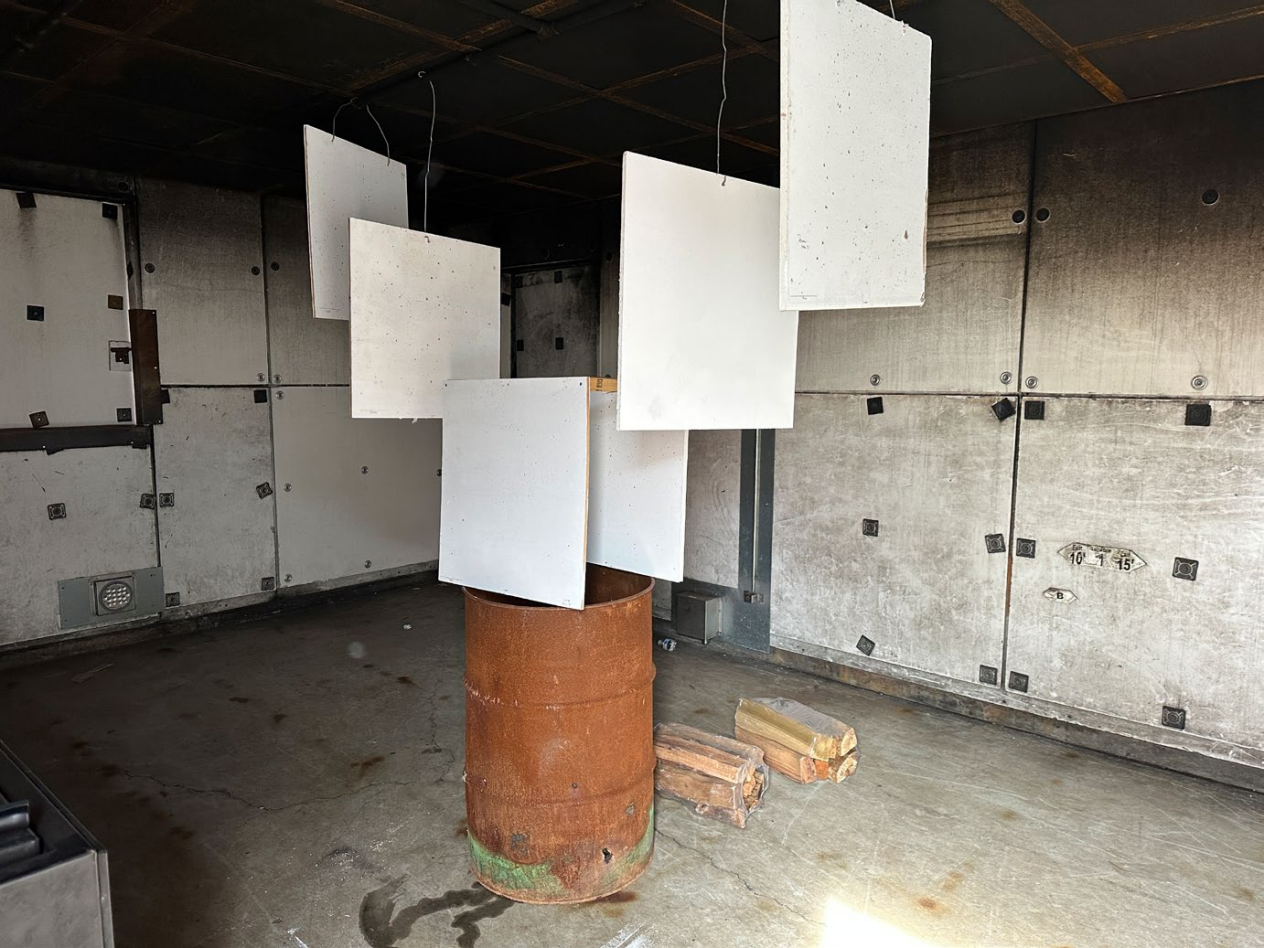
Pattern sample placement relative to the heat source in fire laboratory.

**FIGURE 2:**
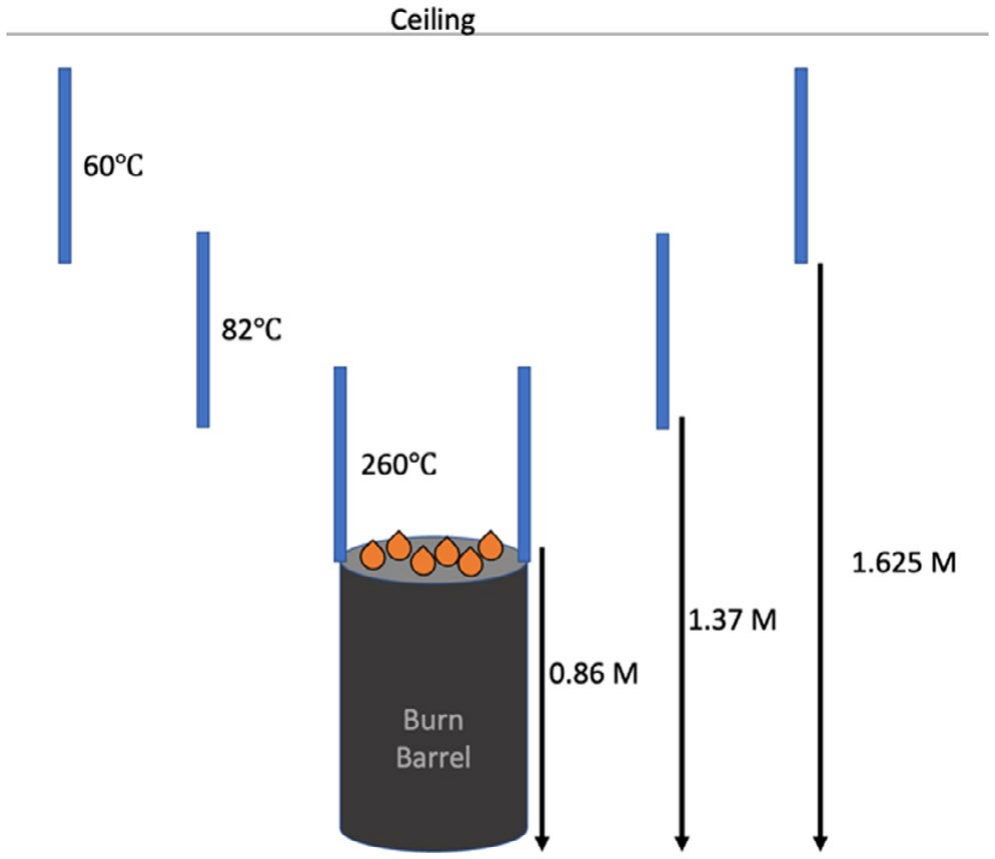
Diagram depicting the pattern sample orientation and measured temperature relative to the heat source.

The impact pattern samples were then transported to the fire laboratory of the Roswell Alpharetta Public Safety Training Center for fire and high heat environment exposure. This is a building that is designed to allow for fire generation and propagation to train firefighters. The 10 stain patterns were suspended over a burn barrel to allow for tiered levels of fire or thermal exposure (Figure 1). Training center staff then ignited the burn barrel, and the fire was allowed to propagate for 5 min, the approximate average response time of a fire company to a fire incident [17]. Prior to the extinguishment of the fire, a Ryobi Infrared Thermometer was used to measure the surface temperatures of the target samples at the different tiers, that data was then recorded (Figure 2). The fire was extinguished with a pressurized water extinguisher and the stain patterns were photographed with metric scales.

### 2.4 Digital imaging

Still image photography was captured using a Fuji XT1‐UV/IR mirrorless camera using three lens variants based on the stain imaging requirement: a Fujinon Aspherical Super EBC *f* = 60 mm 1:2.4 lens, a Fujinon Aspherical Super EBC XF 18–135 mm 1:3.5–5.6 R LM OIS WR lens, and a Fujinon Aspherical Nano‐GI XF 16 mm 1:1.4 R WR lens. The images were captured at the highest resolution setting of “Fine” with the ISO, f‐stop, and shutter speed adjusted to obtain the best photograph for examination. The images were then utilized to calculate scale‐based measurements or incorporated into the computer program Faro Zone 3D Expert (FZ3D) for the area of convergence and origin analysis. In the authors' experience the use of high frame rate digital video imaging aids in the qualitative examination of droplet dynamics on a minute scale. In this study, select bloodstains were captured using a Sony RX10 IV, set to High Frame Rate (HFR) mode, which was recorded at 960 frames per second. Post‐capture analysis was completed using playback software VLC and Adobe Premiere.

### 2.5 Reconstruction methodology

The foundational mathematical relationship in BPA is the ratio between the stain's width and length as a function of the angle at which the blood drop made initial contact with the target surface. This angle is a key step in any reconstruction of bloodstain flight path and is reflected in the angle of impact equation (Equation 1), [18, 19]. This study utilized the FZ3D computer program's bloodstain pattern analysis module to obtain these measurements. The initial step involved importing a photograph of the stains with a metric scale into the “Power Tools: Blood Spatter Analysis” module. The photograph's scale was used to calibrate the image size, and then the ellipse tool was utilized to measure the stain's width and length from its leading edge. These measurements were then recorded in a spreadsheet and applied to the following equation:

$$\text{Angle of Impact}=\text{arcsine}\;\left(\text{width}/\text{length}\right)$$

Or expressed as:

(1) $$\theta =\sin^{-1}\left(\frac{\text{width}}{\text{length}}\right)$$

The process of estimating the AOO was also conducted using the BPA module within FZ3D. This required the import of a scale depicting photograph of the pattern, the selection of the images scale and alignment of the image, and then the ellipse marking selection of 15–20 stains for FZ3D to automatically estimate the AOO. FZ3D then provided X, Y, and Z coordinates of the calculated AOO with related standard deviations.

### 2.6 Results

**FIGURE 3:**
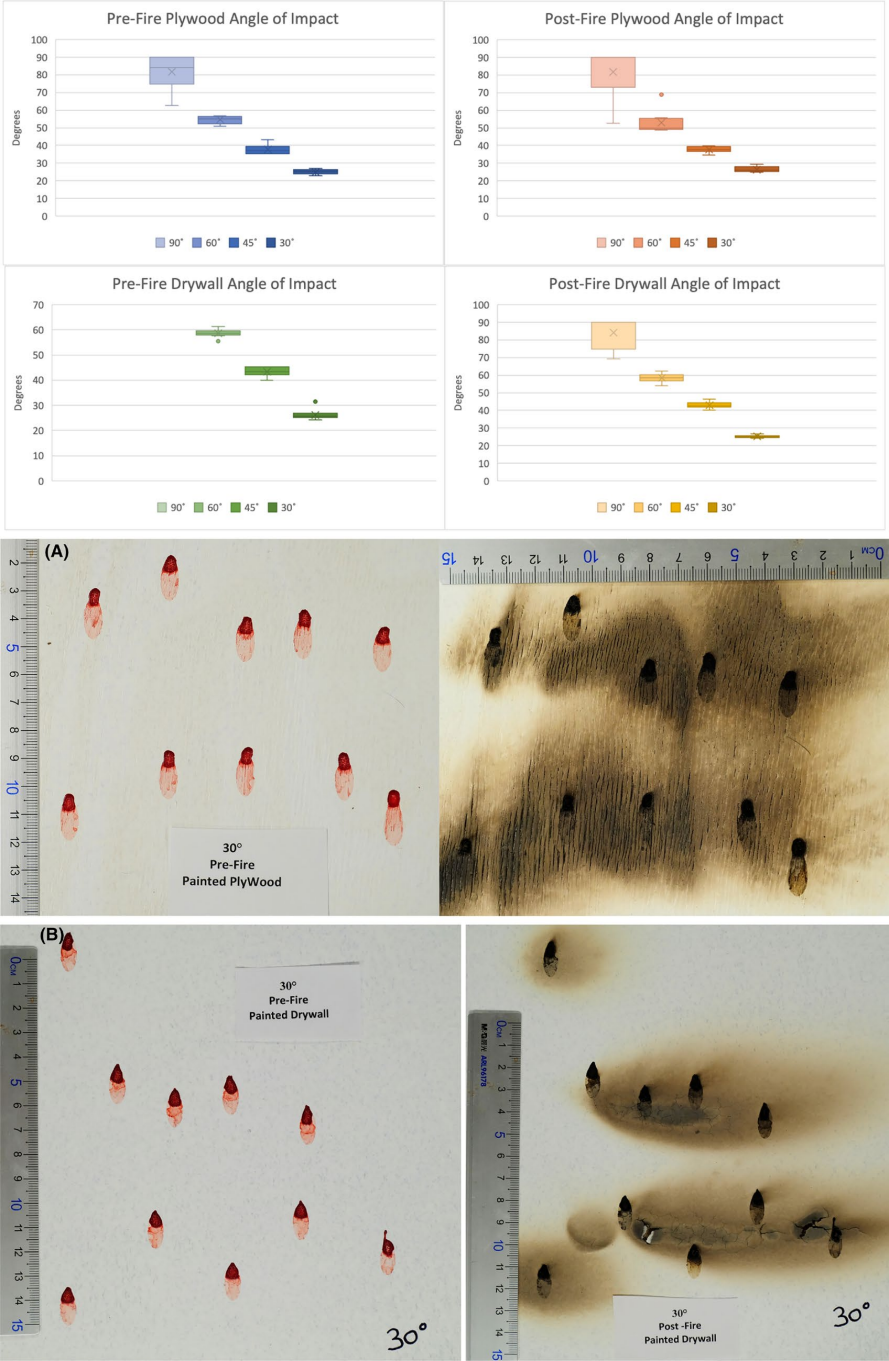
Comparison graph of angle of impact data: Pre‐fire plywood stains (top left), post‐fire plywood stains (top right), pre‐fire drywall stains (bottom left), post‐fire drywall stains (top left) showing the mean (x), range (line bar), and standard deviation (color bar) of calculated angles of impact. (A). Comparison of pre‐fire stains on painted plywood (left) and post‐fire exposed stains (right), for stains with an angle of impact of 30°. (B) Comparison of pre‐fire stains on painted drywall (left) and post‐fire exposed stains (right), for stains with an angle of impact of 30°.

High heat environments and direct exposure to fire present an extreme environmental variable to deposited bloodstain patterns. The physical destruction of stains or the loss of stain material through evaporation leads to unique physical characteristics such as alteration of the stain's original border and internal stain appearance (e.g., discoloration, cracking or flaking, and formation of ligament structures). Figure 3 depicts the comparison of the pre‐fire and post‐fire angle of impact calculations for the drywall and plywood target surfaces. Within the drywall data set, minimal alteration between the pre‐fire and post‐fire exposure stain measurements and angle of impact calculations were observed. Apart from four stains from the 90‐degree set, all others exhibit a standard deviation change of less than 2 degrees. Of the four stains from the 90‐degree set, the fire application had destroyed the original stain boarders and matrix to the point that measurement was unreliable. It should be noted that the absence of any graphic of 90‐degree data on the pre‐fire drywall dataset in Figure 3 (lower left graph) is due to the stains exhibiting no variation and all stains being calculated at 90 degrees. The lack of substantial alteration of measurement is observed across all target sets: drywall, plywood, and glass (Figure 3A,B).

The plywood sample set exhibits a less than two‐degree difference in standard deviation in pre‐ and post‐fire exposure, with the majority being less than one degree. Out of the forty stains on the plywood surface, only two could not be measured post‐fire due to target destruction. This again demonstrates the toughness of stains as the plywood target surface would combust during fire application and would have to be extinguished, which could cause possible dilution complications; however, there were no characteristics of dilution observed.

**FIGURE 4:**
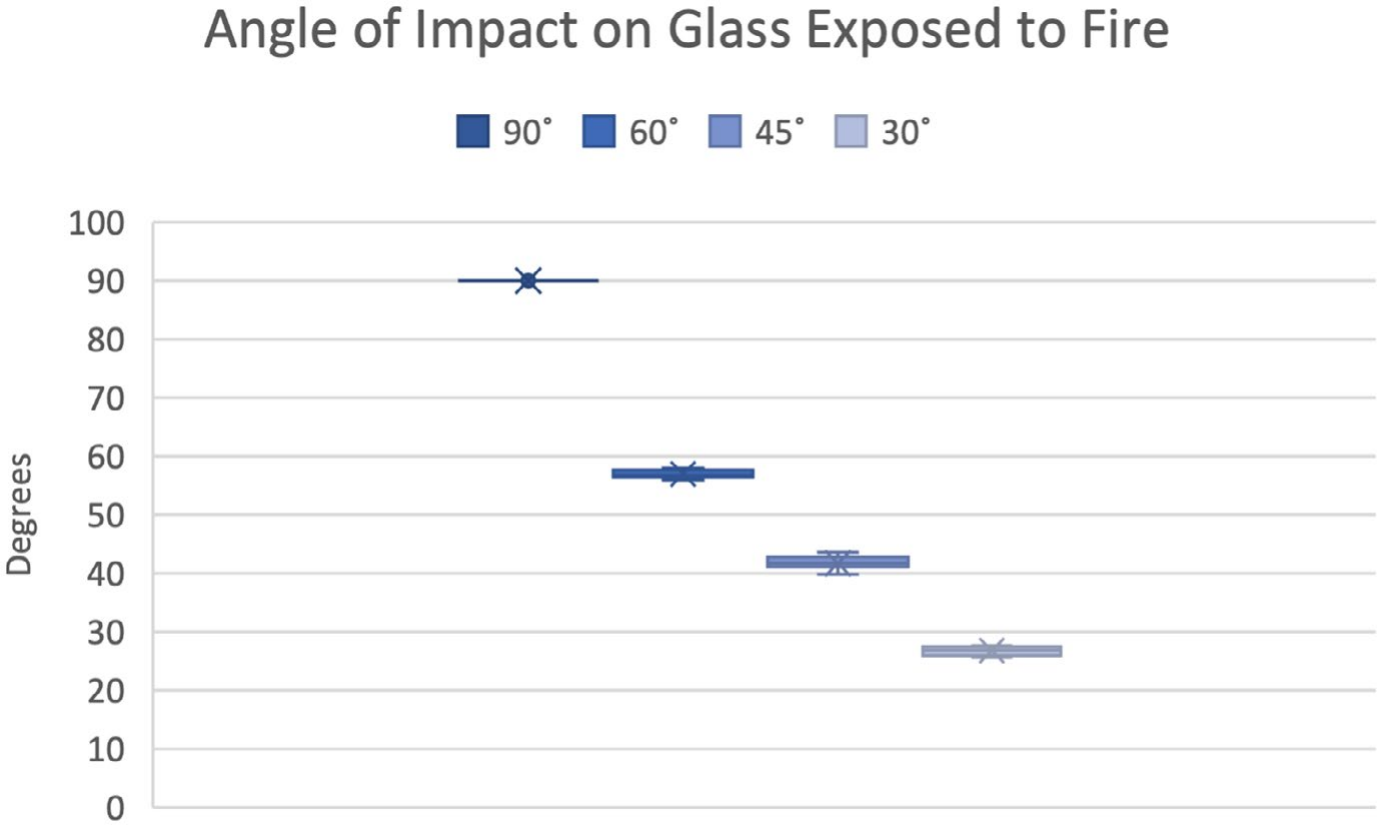
The angle of impact range observed on glass substrate in post‐fire stains showing the mean (x), range (line bar) and standard deviation (color bar) of calculated angles of impact.

The glass trial permitted the isolation of the bloodstain and the effects of fire independent of damage to the target surface. This was visualized using high frame rate video analysis captured under the glass sheet. The glass target permitted complete visualization of the exposed stain and the characteristics occurring during heat exposure. There was no significant discrepancy between the measurement of pre‐ and post‐fire data sets; with the standard deviation ranging from 0 to 2.91 degrees within the examined angle sets (Figure 4).

Within the area of origin estimation, the stain patterns on drywall and wood targets were photographed prior to and after exposure to the high heat environment. Due to the size and structural integrity under direct fire exposure, the glass substrate was not utilized during the AOO experimentation. The images of drywall and wood patterns were submitted to AOO estimation using FZ3D, which provided a mean AOO coordinate in coordinate planes (X, Y, and Z). Within this data, the X position represents the lateral deviation relative to the vertical target, the Y position represents the calculated distance from the area of origin to the target, and the Z position represents the computed height. Table 1 illustrates the results of pre‐ and post‐fire exposure.

**TABLE 1:** Comparison of pre‐ and post‐fire area of origin estimation.

Pre‐Fire exposure area of origin estimation (meters)			
	X‐coordinate	Y‐coordinate	Z‐coordinate
Actual Distance	0	1	1
Calculated Mean	0.03	0.43	1.2
Difference	0.03	0.56	0.2
Standard Deviation	0.04	0.16	0.05

Post‐Fire Exposure Area of Origin Estimation (Meters)			
	X‐Coordinate	Y‐Coordinate	Z‐Coordinate
Actual Distance	0	1	1
Calculated Mean	0.02	0.43	1.18
Difference	0.02	0.56	0.18
Standard Deviation	0.02	0.16	0.05

## 3 DISCUSSION

Fire can affect the appearance of bloodstains in several ways. The presence of a high heat environment and direct fire causes blood to dry, dehydrate, retract, crack, delaminate from the target, and become discolored, all of which can alter the appearance of the stain. Thermal exposure causes the blood to begin to evaporate and become more concentrated, creating darker and internally disfigured stains. The intensity of the fire, its duration, and the type of surface on which the blood is located can also influence the appearance of the stain. However, as long as the stain exhibits visible border characteristics from the original stain, it remains present on the tested surfaces and allows for accurate reconstructive measurements. A comparison of both the angles of impact data sets and the AOO datasets found no statistical difference between the pre‐fire and post‐fire calculations.

**FIGURE 5:**
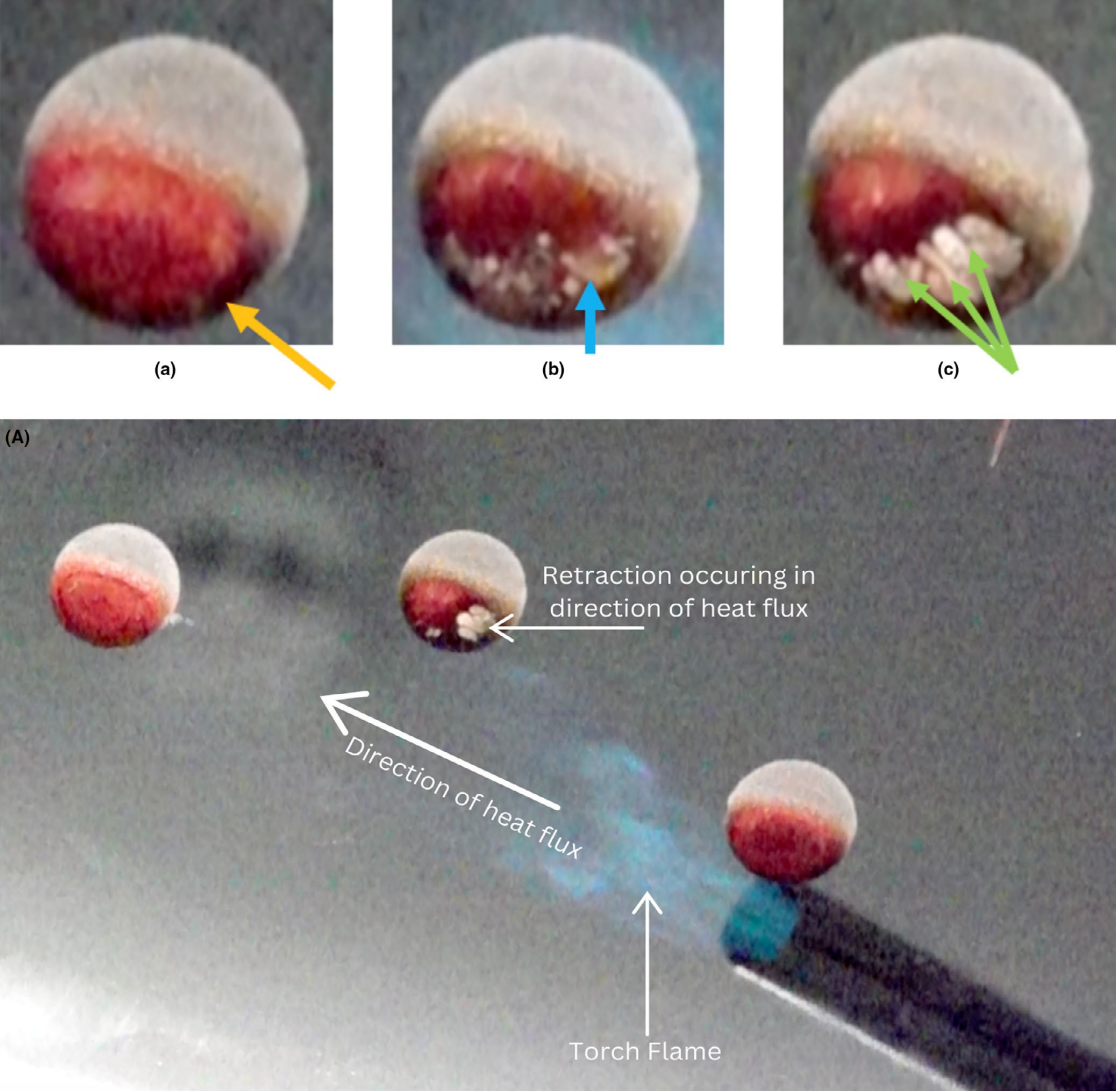
Still frames from highspeed frame rate video of the blood drop's retraction to fire exposure. (Left) (a) Heat Applied and begins to discolor from side in initial contact. (Middle) (b) Rapid dehydration causes retraction to initiate. (Right) (c) Complete thermal retraction in the direction of the applied heat. Leaving behind web‐like characteristics and predominant void in area of initiation. (A) Still frame from highspeed frame rate video of the blood drop's retraction direction in relation to the direction of the heat flux on the glass target.

The stain's alteration process is best understood by high frame rate video analysis through the glass substrate. As the bloodstain is initially exposed to fire, there is an immediate discoloration that begins from the side exposed to the high heat. This is immediately followed by the dehydration of the fluid and retraction of fluid ligaments, forming a web‐like appearance (Figure 5). The web structure corresponds to the direction from which the thermal exposure originates (side of the stain closest to the heat source). In this observation we see that the if the stain were to be bisected, the side of the stain with the absence of blood is the side from which the heat was initially applied (Figure 5A). Detailed examination for characteristics of this retraction in fire incidents may aid the fire investigator in determining the direction or location from which the fire originated. This approximation of the direction of the heat source or fire is of great investigative value. It is another physical characteristic that can be utilized in cause and origin determinations within fire investigations.

**FIGURE 6:**
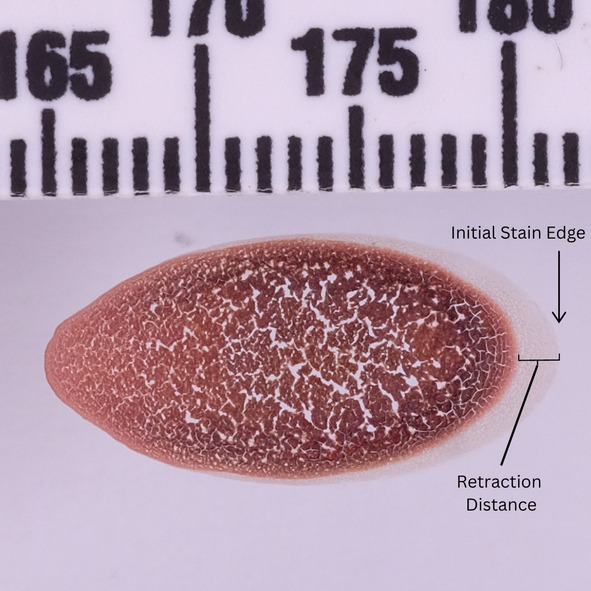
A bloodstain on glass after high heat exposure, depicting the original edge characteristics.

Continuing in the sequence of exposure, prolonged exposure results in further discoloration, vaporization of material, and combustion. Even with material loss, border characteristics remain from the initial stain deposition. Depicted in Figure 6, the initial stain border can be measured even after the retraction phase occurs from thermal exposure. This fossilized edge characteristic is the same that is observed in skeletonized stains that have undergone alteration from an applied wiping motion.

**FIGURE 7:**
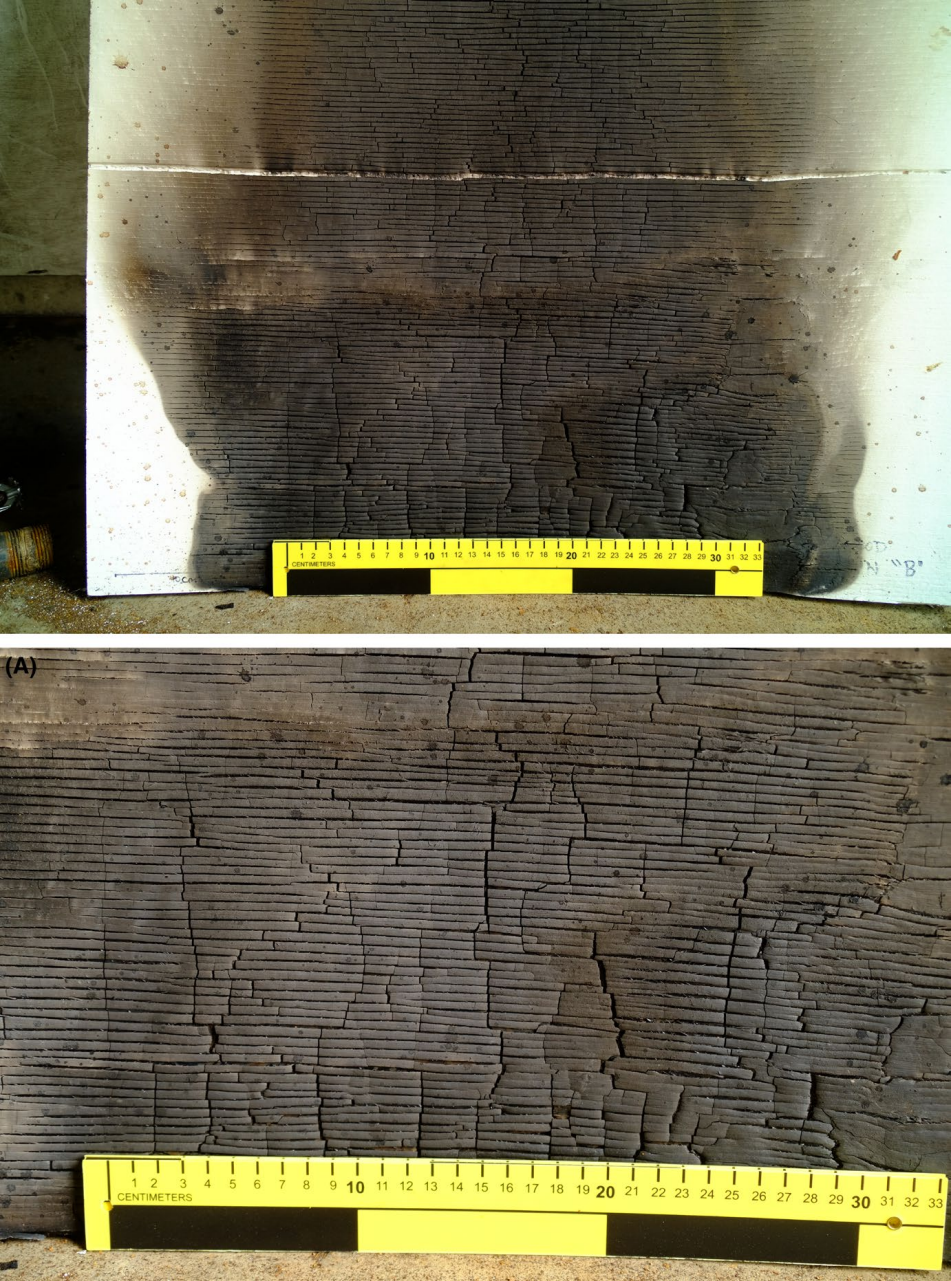
Image depicting heavily damaged impact pattern after exposure to fire, causing charring and alteration to the plywood surface. (A) Close‐up image of bloodstains on the heavily damaged plywood surface yielding the impact pattern.

These observations highlight the resiliency of bloodstains in high heat environments. Examination of the study's most damaged impact patterns target surfaces yielded the presence of bloodstains despite extreme alteration and charring of the target (Figure 7A). The failure to considerably alter or destroy the bloodstains allows the examiner to locate and measure these stains. These measurements can then be used to determine reconstructive components of the stain or pattern. Leading to the conclusion that even after exposure to a destructive force such as fire, the analysis of bloodstain patterns can still reliably estimate the area of convergence and the area of origin.

## 4 CONCLUSION

This study has provided critical insights into the alterations of bloodstain patterns in high heat environments and their implications for forensic investigations. Our research has demonstrated that extreme temperature can significantly alter the physical characteristics of bloodstains; however, characteristics of the original stain are preserved for post‐fire analysis. By systematically examining the changes in bloodstain patterns under various temperature conditions, we have established that fire does not affect the reliability of AOO estimation, provided that the edge characteristics of the stains are visible and that the underlying surfaces are not destroyed or warped by the hot temperatures or fire. Future research should focus on expanding the understanding of how different materials and surfaces react to high heat conditions in the presence of bloodstains. In summary, the findings from this study underscore the importance of considering environmental factors in the analysis of bloodstain patterns at crime scenes. This research enhances the observational knowledge available to BPA analysts, ultimately contributing to the accurate analysis of stain patterns in cases involving fire or high‐temperature environments.

## FUNDING INFORMATION

Dan Rahn Research Grant, funded by the International Association of Bloodstain Pattern Analysts.

## CONFLICT OF INTEREST STATEMENT

The authors have no conflicts of interest to report.

## DECLARATION OF GENERATIVE AI AND AI‐ASSISTED TECHNOLOGIES IN THE WRITING PROCESS

During the preparation of this work, the authors used Open AI ChatGPT4 (San Francisco, California) and Grammarly (San Francisco, California) to improve readability and language syntax. After using this tool/service, the authors reviewed and edited the content as needed and take full responsibility for the content of the publication.

## References

[1] Snyder ML , Aldredge RC . Trial by fire: comparing DNA degradation in blood versus semen after fire exposure. J Forensic Res. 2016;7(5):352. 10.4172/2157-7145.1000352

[2] Hitt C . Man charged with murder, arson after attempting to cover up child's death with explosion. NY Daily News. 2017. https://www.nydailynews.com/2017/12/28/man‐charged‐with‐murder‐arson‐after‐attempting‐to‐cover‐up‐childs‐death‐with‐explosion/ Accessed 26 Nov 2024.

[3] ABC 13 Eyewitness News . California man allegedly kills wife, sets crime scene ablaze. Houston, TX: ABC, Inc.; 2014. https://abc13.com/missing‐person‐escondido‐murrieta‐house‐fire‐freddy‐perez‐rodas/217918/. Accessed 26 Nov 2024.

[4] Bevel T , Gardner RM . Bloodstain pattern analysis with an introduction to crime scene reconstruction. Vol 121. 3rd ed. Boca Raton, FL: CRC Press; 2008. p. 154–155.

[5] Dryzal D . Bloodstain pattern analysis: Applications and challenges. Vol 2. Pittsburgh, PA: D.U.Quark; 2018. p. 22–29. 10.18523/duquark2018.2.2

[6] Sapp AD , Huff TG . Arson‐homicides: Findings from a national study. Washington, DC: U.S. Department of Justice; 1995. NCJ Report No. p. 156232. https://www.ojp.gov/ncjrs/virtual‐library/abstracts/arson‐homicides‐findings‐national‐study Accessed 26 Nov 2024.

[7] Brady T , Tigmo J , Graham G Sr . Extreme temperature effects on bloodstain pattern analysis. IABPA News. 2002;18(2):3–20.

[8] Larkin BAJ , Banks CE . Preliminary study on the effect of heated surfaces upon bloodstain pattern analysis. J Forensic Sci. 2013;58(5):1289–1296. 10.1111/1556-4029.12165 23865610

[9] Vineyard AR , Hazelrigg EJ , Ehrhardt CJ , Connon CC . Evaluation of Bluestar® forensic magnum and other traditional blood detection methods on bloodstained wood subjected to a variety of burn conditions. J Forensic Sci. 2019;64(3):878–887. 10.1111/1556-4029.13959 30380138

[10] Tontarski KL , Hoskins KA , Watkins TG , Brun‐Conti L , Michaud AL . Chemical enhancement techniques of bloodstain patterns and DNA recovery after fire exposure. J Forensic Sci. 2009;54(1):37–48. 10.1111/j.1556-4029.2008.00911.x 19018938

[11] AAFS Standards Board . ASB technical report 033: terms and definitions in bloodstain pattern analysis. AAFS Standards Board. 2017;1–4. https://www.aafs.org/sites/default/files/media/documents/033_TR_e1_2017.pdf Accessed 26 Nov 2024.

[12] Christman DV . A study to compare and contrast animal blood to human blood product. Everett, Washington: Snohomish County medical Examiner's office; 1997. https://static1.squarespace.com/static/543841fce4b0299b22e1956a/t/54be91a3e4b054556ec58df3/1421775267210/Study+to+Compare+Animal+and+Human+Blood+Christman+1997.pdf Accessed 26 Nov 2024.

[13] Spivey DG . Effect of wind on region of origin calculations of impact bloodstain patterns: implications for bloodstain pattern analysis [dissertation]. Perth, Australia: University of Western Australia; 2016.

[14] Miles H . Bloodstain pattern analysis: developing quantitative methods of crime scene reconstruction through the interpretation and analysis of environmentally altered bloodstains [dissertation]. London, UK: University College London; 2014.

[15] Raymond M , Smith ER , Liesegang J . The physical properties of blood‐forensic considerations. Sci Justice. 1996;36(3):153–160. 10.1016/S1355-0306(96)72585-3 8789932

[16] Brownson DAC , Banks CE , El‐Sayed M , Brownson DAC , Banks CE . Crime scene investigation II: the effect of warfarin on bloodstain pattern analysis. Anal Methods. 2011;3(7):1521. 10.1039/clay05196b

[17] U.S. Fire Administration/National Fire Data Center . Structure fire response times. Topical Fire research series. Vol 5. Emmitsburg, MD: U.S. Department of Homeland Security; 2006. https://apps.usfa.fema.gov/downloads/pdf/statistics/v5i7.pdf Accessed 26 Nov 2024.

[18] Balthazard V , Piedelievre R , Desoille H , Derobert L . Study of drops of projected blood. Paris, France: XXII Congress of Forensic Medicine; 1939. p. 265–323.

[19] Rizer C . Police mathematics; a textbook in applied mathematics for police. Springfield, IL: Charles C Thomas; 1955.

